# Transgenerational dynamics of gut microbiota in black soldier fly larvae (*Hermetia illucens*) reared on a novel substrate

**DOI:** 10.1128/spectrum.01903-25

**Published:** 2026-03-31

**Authors:** Shaktheeshwari Silvaraju, Amber Lim Ching Han, Tang Yong Jen, Sandra Kittelmann, Nalini Puniamoorthy

**Affiliations:** 1 Department of Biological Sciences, National University of Singapore 145755 , Singapore, Singapore; 2 Wilmar International Limited 245010, Singapore, Singapore; Shandong University, Qingdao, China

**Keywords:** genetic, gut microbiota, transgenerational, *Hermetia illucens*, black soldier fly, core microbiota, Sloan neutral model, selective breeding, adaptation

## Abstract

Understanding the transgenerational dynamics of gut microbiota in black soldier fly larvae (BSFL) is essential for optimizing their performance on novel waste substrates in industrial settings. In this study, a wild-type BSF population was divided into six sub-lines and reared over four generations: one line on standard chicken feed (CF) and five on a novel plant-based diet (WIL), four of which were subjected to selection for increased larval size. Despite a shared genetic origin, sub-lines exhibited divergent trajectories in both larval weight and gut bacterial composition. Larval weight increased up to the second (G2) or third (G3) generation but declined sharply at generation four (G4) across all lines. Parent–offspring regressions revealed low narrow-sense heritability, suggesting limited genetic contribution to larval weight variation. Gut microbiota analysis revealed that early developmental stages were most sensitive to generational shifts, with the transition from G3 to G4 showing the strongest changes in community structure. Core species, including multiple *Enterococcus* spp. and *Providencia rettgeri*, were consistently detected across all lines, diets, and generations. Most of these were classified as “Neutral” or “Above” in the Sloan Neutral Model, suggesting functional and survival importance regardless of selection regime or substrate. Deviations from neutrality were more pronounced in WIL-fed lines, consistent with intermediate disturbance dynamics, while CF-fed lines exhibited fewer deviations under low-disturbance, stable conditions. Notably, growth-associated taxa, such as *Bacillus* and *Paenibacillus*, peaked in G2 to G3 but declined at G4, whereas immune modulatory taxa, such as *Klebsiella*, increased in abundance. These trends indicate a shift from growth-promoting to digestion- and resilience-oriented microbial strategies under prolonged dietary stress. The stochastic emergence of distinct microbial patterns across sub-lines underscores the plastic and adaptable nature of BSFL gut microbiota. Recognizing this plasticity emphasizes the need to maintain large, genetically and microbially diverse BSF populations, which is critical for preserving functional stability and supporting long-term adaptation when utilizing novel or suboptimal substrates in industrial production.

**IMPORTANCE:**

The black soldier fly is rapidly gaining recognition globally as a key agent in circular bioeconomy for its ability to convert diverse organic waste into high-value products. However, the long-term stability and resilience of its gut microbiota on novel, low-cost diets remain poorly understood. This study addressed that knowledge gap by tracking transgenerational changes in larval gut microbial communities over four generations, using a single population reared on a novel diet, both with and without selection for larval size. Despite a shared genetic background, different sub-lines developed distinct microbiota and growth patterns, with early developmental stages showing the greatest sensitivity to generational microbial shifts. Initial increases in certain bacterial groups were followed by community restructuring by the fourth generation, indicating a dynamic but unstable microbial response to prolonged dietary stress. These findings highlight the importance of preserving microbial and genetic diversity when breeding black soldier flies for industrial use. Understanding how host-microbiota responses shift across generations is essential for sustaining performance and ensuring resilience in large-scale black soldier fly production systems.

## INTRODUCTION

The global rise in food loss and agricultural by-product accumulation presents urgent environmental and economic challenges. An estimated 1.5 billion tons of food waste are generated annually, contributing to 8–10% of global greenhouse gas emissions and resulting in nearly USD 1 trillion in economic losses (1). Furthermore, the processing of agricultural commodities generates vast quantities of underutilized waste side-streams, such as palm kernel meal (over 7 million tons annually) (2) and okara, the fibrous residue of soy milk and tofu production (estimated at 14 million tons per year) (3).

Insect-based bioconversion, specifically using the black soldier fly (*Hermetia illucens*; BSF), provides a sustainable solution for valorizing these waste streams. Black soldier fly larvae (BSFL) efficiently convert diverse organic substrates into high-value outputs, including protein, lipids, and frass, positioning them as key agents in the circular bioeconomy (4, 5). Consequently, commercial interest in BSFL has expanded rapidly, with industrial-scale rearing facilities established globally (6, 7).

Optimizing BSFL production pipelines requires a deep understanding of biological processes involved in waste bioconversion. At the pre-valorization stage, genetic background and diversity influence larval adaptability and overall performance (8–11). At the valorization stage, both host genetics and diet composition are known to modulate larval growth, nutritional profiles, and gut microbiota composition (12–15). However, most investigations are limited to a single generation, offering only a static snapshot of host–microbe interactions in response to specific diets.

Emerging evidence suggests that BSFL can rapidly adapt to novel or suboptimal diets. Transgenerational effects have been linked to improvements in larval biomass, feed conversion efficiency, and oviposition performance (16–18). While these changes are often considered genetically driven, interacting factors, such as diet composition (e.g., micronutrients, porosity, particle size) and the gut microbiota, may synergistically shape adaptive phenotypes (19, 20). Studies on selective pressure in insects, such as energy allocation trade-offs in *Musca domestica* and gene expression shifts in BSFL, suggest that adaptation can involve physiological compromises. However, the influence of such trade-offs on gut microbial dynamics over successive generations remains largely unexplored (21–23).

Understanding how the gut microbiota responds to prolonged exposure to low-cost, novel substrates is critical for maintaining performance and consistency in industrial BSFL production. Insights can be drawn from selective breeding research, which inherently spans multiple generations and explores microbial and host co-adaptation. For instance, BSFL selectively bred for cold tolerance (16°C) over nine generations developed a gut microbiota enriched with *Acinetobacter*, *Pseudochrobactrum*, *Enterococcus*, *Comamonas*, and *Leucobacter*. Functional metagenomics in this line revealed the upregulation of pathways related to energy and nutrient metabolism, including amino acid and lipid biosynthesis (24). Similarly, a heat-tolerant line (40°C) exhibited gut microbiota dominated by *Campylobacter*, *Enterococcus*, *Dysgonomonas*, and *Proteus*, likely facilitating enhanced substrate breakdown and energy acquisition (25, 26).

While selective breeding remains a powerful tool, it is resource-intensive for nascent insect production systems. The present study explored transgenerational gut microbiota dynamics in BSFL subjected to targeted selection for increased larval size on a novel waste substrate (WIL diet, a 50:50 blend of palm kernel meal and okara). By using a multi-line selection experiment from a single genetic population, both phenotypic shifts in larval weight and gut microbial community composition were tracked over four generations. This framework was designed to test whether host traits, such as body weight under a novel diet, drive successive changes in the gut microbial communities. The findings aim to shed light on the transgenerational stability and adaptability of the BSFL microbiota, providing implications for scalable, diet-based breeding strategies in industrial settings.

## RESULTS

### Heritability

To evaluate the heritability of pupal weight in BSFL reared on a novel diet (WIL diet), the average progeny pupal weights were regressed against mid-parent pupal weights across eight mating cages. Linear regression analysis yielded a narrow-sense heritability estimate (*h^2^*) of 0.206, based on the slope of the fitted trendline (Fig. S1). The coefficient of determination (*R*^2^) was 0.130, indicating that variation in mid-parent weight explained approximately 13% of the variation in progeny pupal weight.

Although this *h^2^* estimate was modest, the presence of heritable variation in pupal weight suggested that the trait had the potential to respond to selection on the novel, WIL diet. This provided the rationale for initiating large-scale selective breeding experiments to investigate the relationship between selection for larval performance and concurrent gut microbiota dynamics over successive generations.

### Gut bacterial diversity influenced by diet and temporal effects over generations

Gut bacterial composition was analyzed across all sub-lines to evaluate the combined effects of diet, selection, and generation. The wild-type (WT) population was maintained for four generations (G1–G4) and divided into six sub-lines: one control line reared on standard chicken feed (CF) and five lines reared on a novel plant-based diet (WIL1–WIL4 and WILC). WIL1–WIL4 were subjected to selection for increased larval size, while WILC served as the unselected dietary control. A total of 9.71 million raw reads were obtained from high-throughput sequencing using ONT technology. After base calling, demultiplexing, and quality filtering, the final data set contained 2.81 million reads, with an average sequence yield of 15,424 ± 5,533 reads per sample (mean ± SD).

Significant differences in Shannon α diversity were observed across sub-lines (*P* = 0.0011) as well as larval age (*P* = 2.21 × 10⁻¹¹), indicating that these factors were major contributors to gut microbiota diversity. While generation alone did not significantly affect α diversity (*P* = 0.0893), significant interactions between sub-lines and larval age (*P* = 0.0014) and between generation and larval age (*P* = 0.0001) demonstrated the dynamic influence of selection and generational changes on bacterial diversity modulated over time.

**Fig 1:**
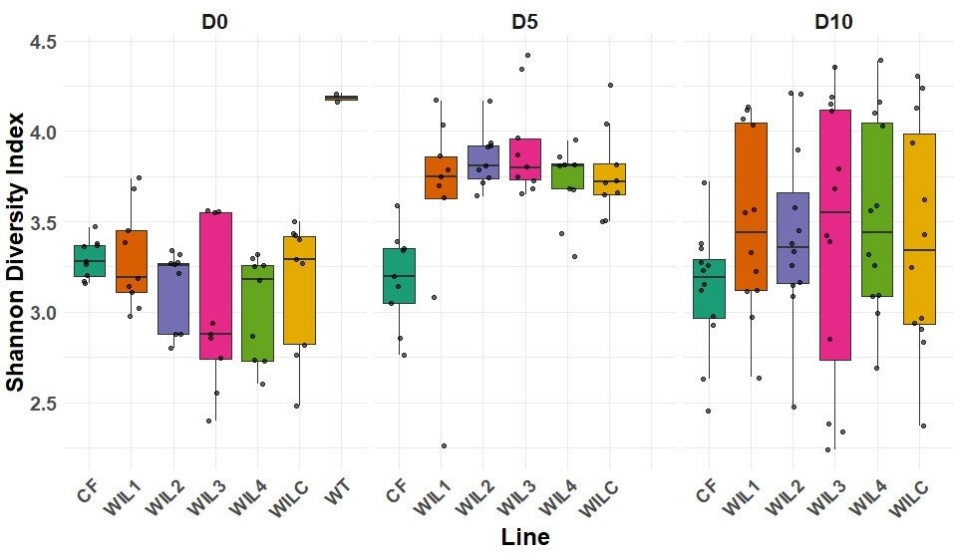
Shannon diversity index comparing bacterial communities across selectively bred sub-lines (CF, WIL1, WIL2, WIL3, WIL4, and WILC) at different experimental days (D0, D5, D10). WT D0 samples represented the initial population from which the sub-lines were derived.

Pairwise Wilcoxon comparisons revealed a strong temporal effect, with Shannon diversity at experimental day 5 (D5) consistently higher than at day 0 (D0) across multiple sub-lines. Specifically, D5 samples of sub-lines WIL2, WIL3, WIL4, and WILC all showed significantly greater bacterial diversity compared to their respective D0 counterparts (all *q* < 0.01; Fig. 1). Conversely, bacterial diversity remained stable across days and generations in CF-fed larvae (*q* > 0.05 for all comparisons). The increased diversity in sub-lines undergoing artificial selection (WIL2-WIL4; WILC) versus the control CF line highlights the dietary pressures exerted by the novel diet in shaping the gut microbiota. However, this effect was not apparent in WIL1, whereby bacterial diversity neither differentiated within the line across experimental days nor between other lines (*q* > 0.05 for all comparisons).

### Rapid adaptation and diversification of gut microbiota over four generations

**Fig 2:**
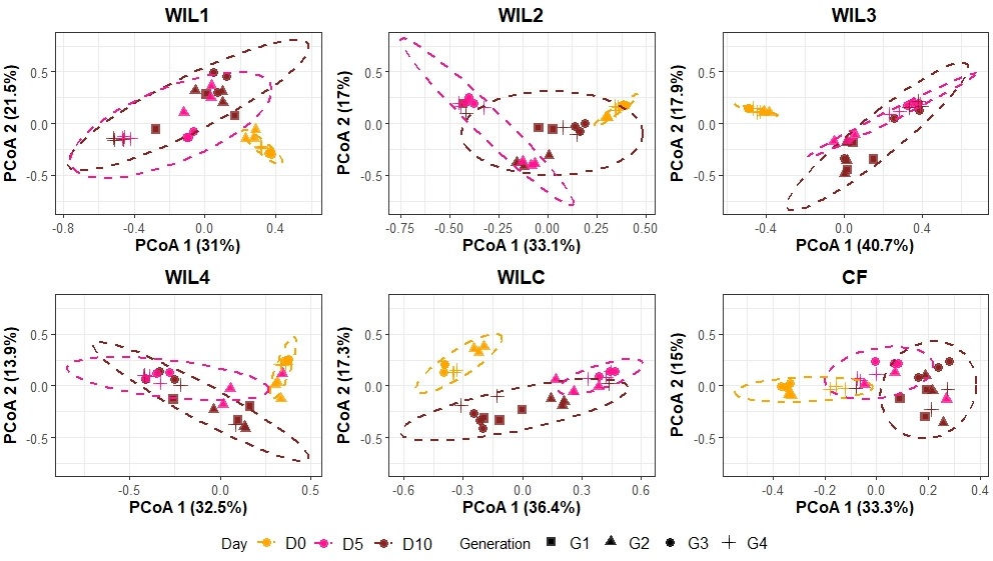
Principal Coordinate Analysis of the bacterial communities at the species level based on Bray–Curtis dissimilarity. Each panel represents different selectively bred sub-lines (WIL1, WIL2, WIL3, WIL4) and control lines (WILC and CF). The data points are colored according to experimental day (D0, D5, and D10) and shaped according to generation (G1 to G4). Ellipses indicate 95% confidence intervals for respective experimental day within each sub-line.

Principal coordinates analysis (PCoA) based on Bray–Curtis dissimilarity at the species level revealed distinct clustering patterns associated with time points and generations (Fig. 2), which were further evaluated with pairwise PERMANOVA.

Across all populations, a strong temporal effect was evident, particularly between early and later time points. In every line, significant differences were observed between D0 and D5 (*q* = 0.004–0.006), confirming that substantial microbial restructuring occurs during this early developmental window. For instance, WIL2 (*q* = 0.006), WIL3 (*q* = 0.006), WIL4 (*q* = 0.006), WILC (*q* = 0.004), and CF (*q* = 0.006) all showed this D0 vs D5 divergence. Significant differences were also detected between D0 and D10 in most lines, reflecting a temporal restructuring of the microbial community at later time points.

However, comparisons between D5 and D10 revealed a less consistent pattern. Some sub-lines, including WIL2 (*q* = 0.032), WILC (*q* = 0.004), and CF (*q* = 0.012), displayed significant differences, while others did not. This suggests that microbial communities reach a plateau or stabilize by mid- to late-adulthood in some lines.

In contrast to the consistent temporal patterns, generational effects were more selective and largely dependent on the specific sub-line and the age. Significant generation-level differences were observed only in artificially selected lines (WIL1, WIL2, and WIL4). For instance, WIL2 showed multiple significant generational shifts (G1 vs G2, G2 vs G3, and G2 vs G4; all *q* = 0.048), indicating a gradual change in community structure across generations. WIL1 showed a change across G2 and G4 (*q* = 0.018), while WIL4 showed a generational effect between G2 and G3 (*q* = 0.036).

To confirm the validity of PERMANOVA outcomes, beta dispersion analysis was performed for all significantly different group comparisons. The majority showed no significant differences in dispersion, indicating that observed compositional shifts were not driven by heterogeneity in within-group variability. However, a few comparisons (e.g., WIL1: D0 vs D5, D0 vs D10) exhibited significant dispersion suggesting within-group variability potentially driven by generational differences and were omitted from interpretation.

No significant generation-level differences were detected in the control lines (CF and WILC; not subjected to larval size selection), reinforcing that the selective breeding process, when applied alongside the novel diet, introduced microbial divergence across generations that was otherwise absent under stable, unselected conditions.

### Differential adaptation within a genetic population

**Fig 3:**
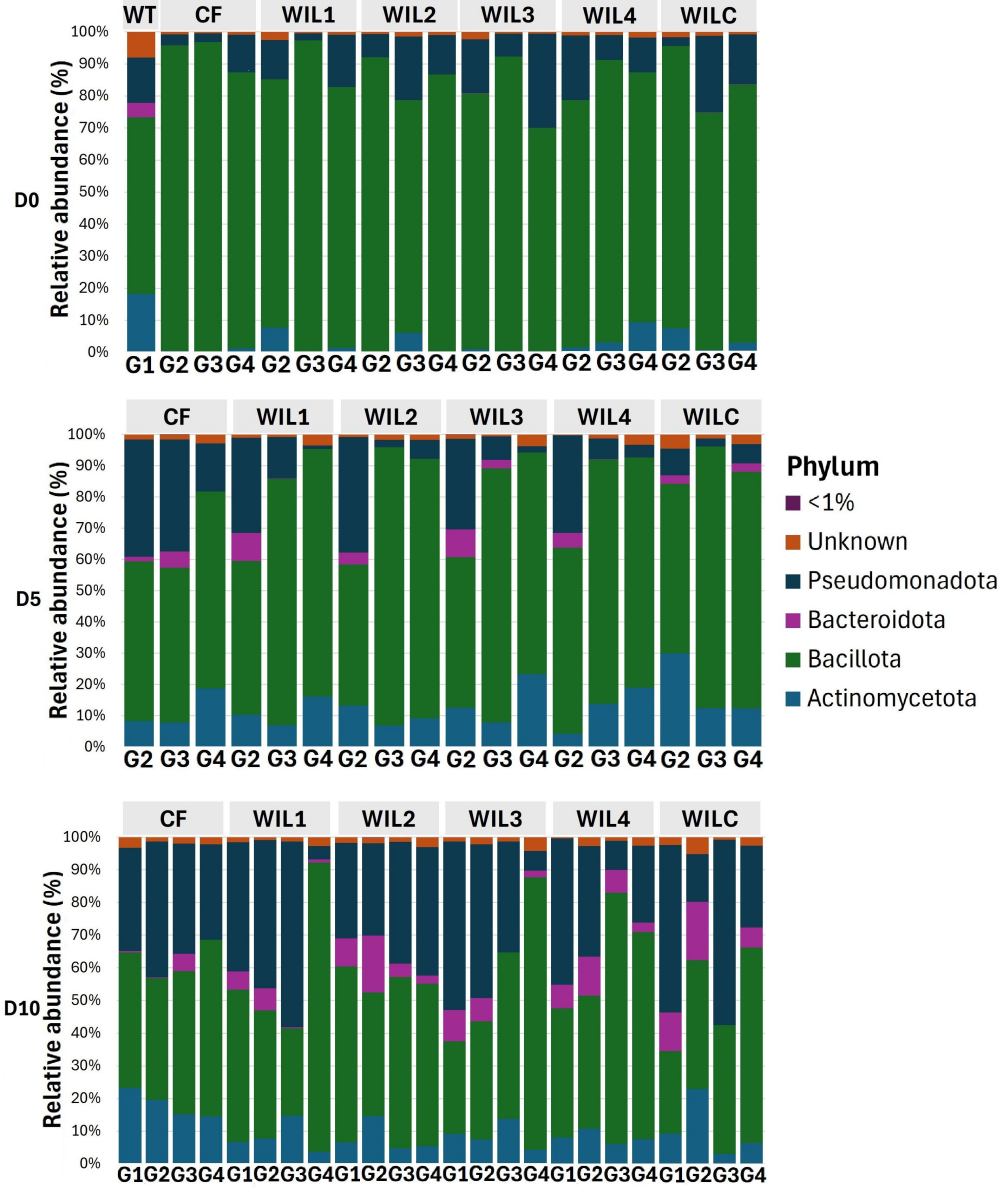
Mean relative abundances of bacterial phyla in the gut of BSFL under selection for larval weight (WIL1–WIL4) on the novel WIL diet, as well as in control sub-lines (CF: standard diet control; WILC: novel diet control). WT represents G1, D0 samples. Relative abundances are shown for days D0, D5, and D10 across generations G1–G4. “<1%” represents taxa with <1% relative abundance, and “Unknown” represents unassigned taxa.

Microbial community structure was profiled at the phylum and genus levels at three developmental time points (D0, D5, and D10) across all sub-lines and generations (Fig. 3). At D0, Bacillota and Actinomycetota dominated across all lines. By D5, WIL lines exhibited a transient enrichment of Bacteroidota exclusively in G2, accompanied by a concurrent increase in Pseudomonadota, a shift not retained in later generations. At D10, Pseudomonadota and Bacteroidota generally increased in both CF and selected WIL lines, though abundances remained variable. Bacillota increased in G4 of WIL1 and WIL3 and in G3 of WIL4, while Bacteroidota was nearly absent in G2 of WIL1, WIL3, and WILC, indicating temporal and generational shifts in community composition driven by selection and dietary adaptation.

**Fig 4:**
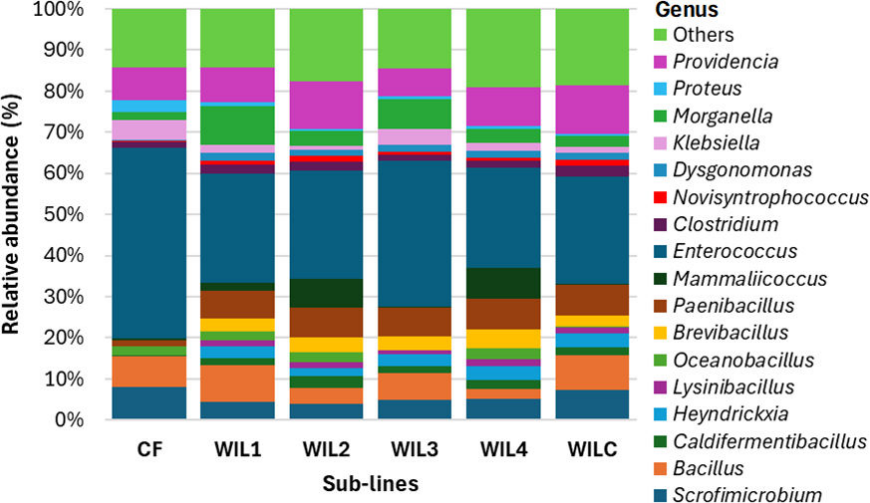
Mean relative abundances of gut-associated genera across all generations, time points, and replicates in BSFL reared under targeted selection for larval size (WIL1 to WIL4) on a novel WIL diet, alongside unselected controls (CF and WILC). “Others” represents all remaining taxa, including those with < 1% relative abundance in the samples and unassigned taxa.

Differential abundance analysis at the genus level using ALDEx2 highlighted significant differences between the CF control and the WIL-fed lines (WIL1 to WIL4 and WILC; Fig. 4). Genera such as *Brevibacillus*, *Caldifermentibacillus*, *Heyndrickxia*, *Lysinibacillus*, and *Paenibacillus* were significantly enriched in all WIL-fed lines compared to CF (*q* < 0.05). In contrast, *Enterococcus* was significantly enriched in CF across all comparisons (*q* < 0.05). Several other genera exhibited more specific patterns. *Proteus* was nearly twice as abundant in CF than in most WIL lines (2.8 ± 3.5% vs 0.4 to 0.8 ± 0.7 to 1.6%), except WIL1, while *Klebsiella* showed higher abundance in CF compared to WIL2 (4.9 ± 5.3% vs 0.9 ± 1.9%; *q* = 0.028). In contrast, *Novisyntrophococcus* was significantly depleted in CF relative to WILC (0.4 ± 2.1% vs. 1.4 ± 2.4%; *q* = 0.026). No significant differences in abundance at the genus level were observed between any of the WIL-fed lines (*q* > 0.05).

### Temporal changes in the gut bacterial community within each generation

**Fig 5:**
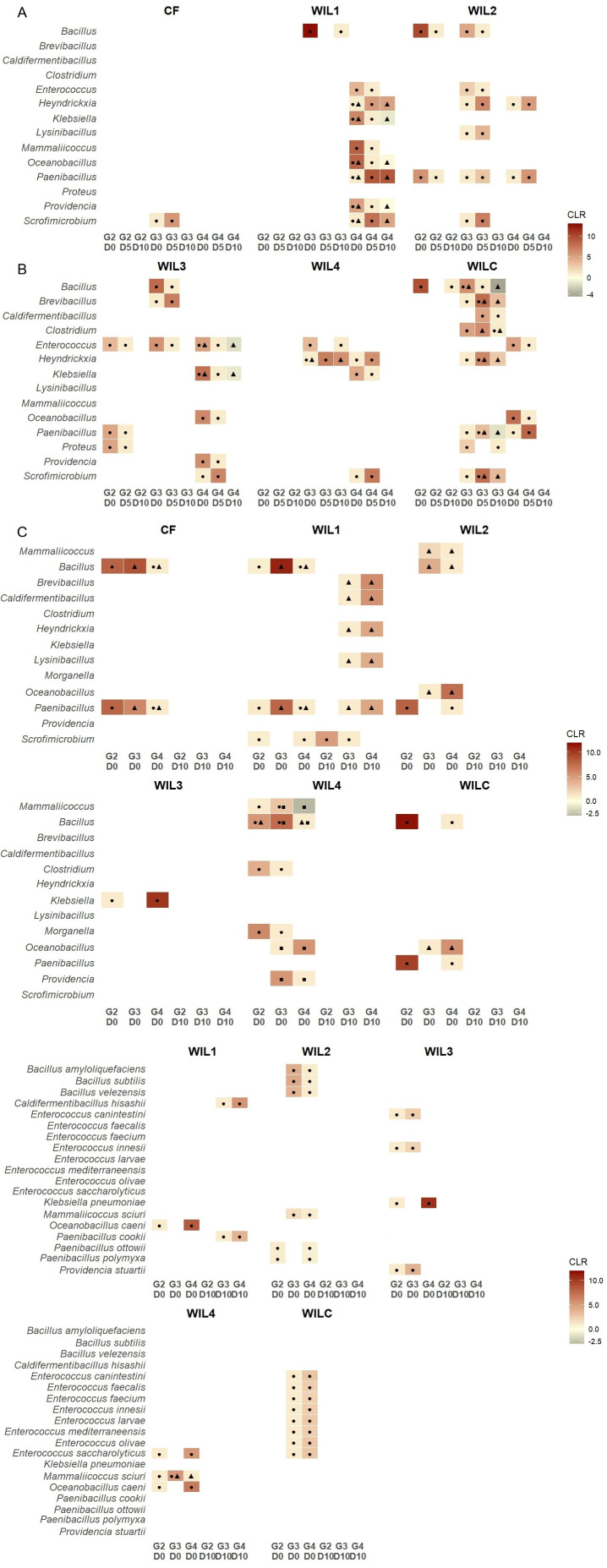
Heatmaps displaying centered log-ratio (CLR) transformed relative abundance of bacterial taxa that were differentially abundant in the gut of BSFL across generations and/or time points in each sub-line. Symbol annotations (● circle, ▲ triangle, ■ square) denote statistically significant pairwise differences (*q* < 0.05). CLR values are color-scaled from black (low abundance) to red (high abundance), with yellow representing CLR ≈ 0 (indicating *q* > 0.05). Only genera with a relative abundance of >1% across all samples were included in the analysis. (**A**) Genera that were differentially abundant between D0, D5, and D10 within each generation (G2 to G4; G1 was omitted due to no significance detected) for each sub-line. (**B**) Genera that were differentially abundant between generations (G2 to G4) within each age group (D0 and D10; D5 was omitted due to no significance detected) for each sub-line. (**C**) Species level differences based on genera that were identified in (**B**); CF was omitted due to no significance detected.

Although no significant genera were detected between the targeted selection lines, several taxa were differentially abundant across ages within each generation (Fig. 5A), highlighting temporal changes in the gut bacterial community. Overall, the CF line displayed a relatively stable community across ages over generations, with only *Scrofimicrobium* differing between D0 and D5 in the third generation (G3: 7.4 ± 2.4% vs. 13.4 ± 8.5%; *q* = 0.048).

Most temporal changes occurred in the WIL lines, particularly in G3 and G4, and were most pronounced between D0 and D5. *Enterococcus* was consistently more abundant at D0 than at D5 across all WIL-fed lines (*q* < 0.05 for all), except for WIL4, where it differed between D0 and D10 (*q* < 0.05). In contrast, several other genera showed significantly higher abundances at D5 and were detected in the majority, though not all, of the WIL-fed lines. For example, *Heyndrickxia* and *Scrofimicrobium* generally peaked at D5 compared to D0 or D10, suggesting transient enrichment at mid-developmental stages. *Paenibacillus* exhibited variable patterns across lines, being higher at D5 in some (e.g., WIL1, WIL2, and WILC) and at D0 in others (WIL2 and WIL3), although the latter pattern was observed only in G2. *Klebsiella*, on the other hand, tended to be more abundant at D0 (*q* < 0.05).

In contrast to the targeted selection lines, the control line (WILC) displayed the greatest number of changes in gut bacterial composition, particularly in G3. Notably, *Caldifermentibacillus* and *Clostridium* were uniquely detected in WILC G3, both showing significant differences between D5 and D10 (*q* < 0.05), with *Clostridium* additionally differing between D0 and D10 (*q* = 0.037). These shifts indicate that, even under relaxed selection, the gut bacterial community structure remained dynamic.

### The shift of specific taxa across generations

Generation-specific dynamics at different developmental stages showed that most shifts in specific taxa occurred primarily at D0, suggesting that gut bacterial restructuring in response to the diet began early in development (Fig. 5B). *Bacillus* and *Paenibacillus* were detected as differentially abundant in all lines, except WIL3 and WIL4 (*q* < 0.05 for all). Among the lines where these genera were detected, *Bacillus* showed its highest abundance in G3 for most lines, whereas in WILC, it peaked earlier, in G2. *Paenibacillus* was generally most abundant in G2, except in WIL1, where it peaked in G3. *Klebsiella* was the sole taxon showing generational differences in WIL3, being more abundant in G2 than in G4 (2.5 ± 1.8% vs 0.6 ± 0.4%; *q* = 0.022). Generational variation at D10 was observed only in WIL1, where *Brevibacillus*, *Caldifermentibacillus*, *Heyndrickxia*, *Lysinibacillus*, and *Paenibacillus* were more abundant in G4, whereas *Scrofimicrobium* was higher in G2 (*q* < 0.05 for all).

WIL4 showed the highest number of differentially abundant taxa across generations at D0, potentially driven by generational shifts in the early colonizers of the gut. Specifically, *Morganella* and *Providencia*, which were not differentially present in any other sub-lines, were significantly higher in WIL4 G2 individuals compared to G3 (12.4 ± 20.3% vs 1.4 ± 1.8%; *q* = 0.044) and in G3 individuals compared to G4 (1.8 ± 0.8% vs 0.8 ± 0.4%; *q* = 0.026) respectively.

### Correlation between larval weight and gut microbiota

**Fig 6:**
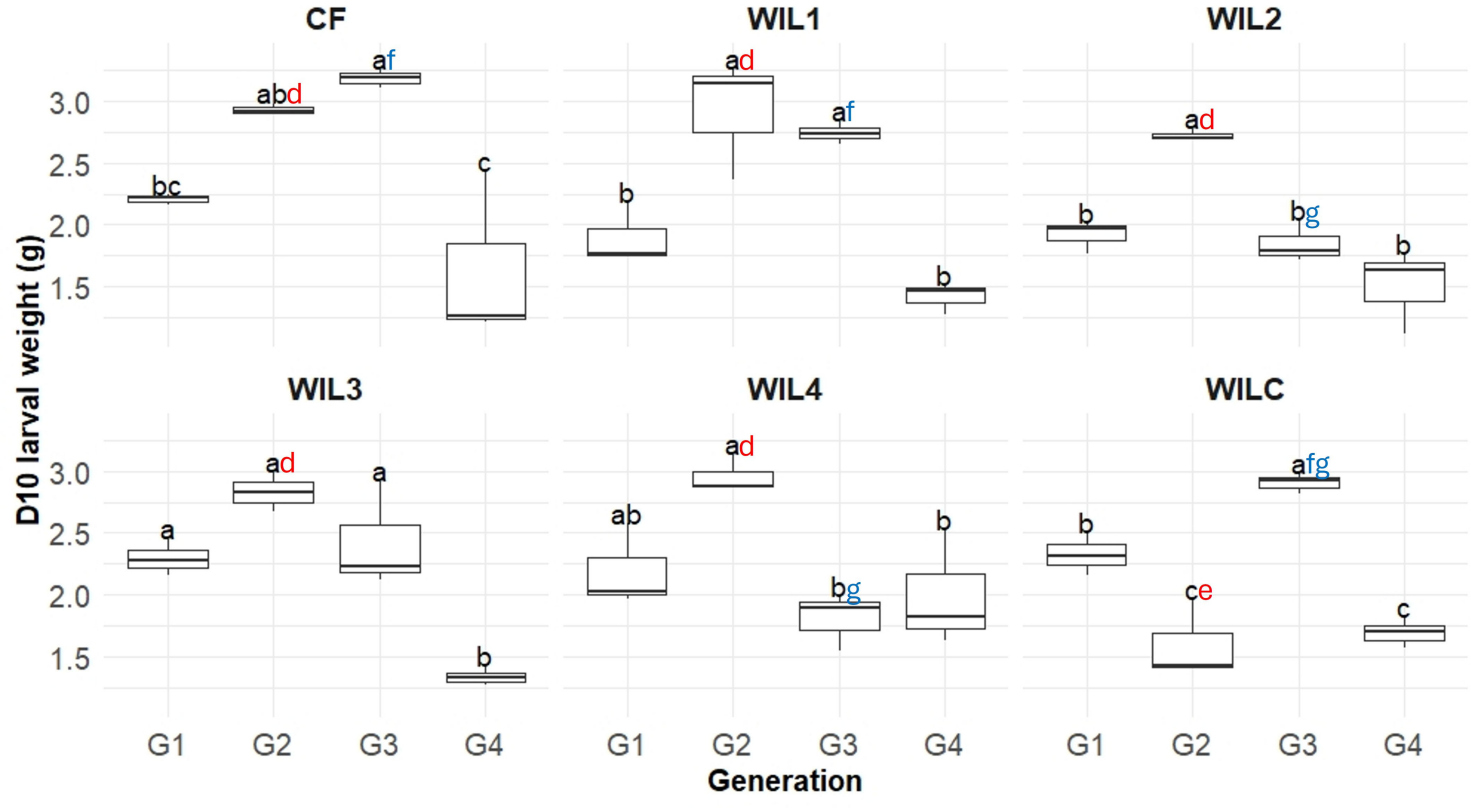
Boxplots showing day 10 larval weight (g) of 10 larvae collected across four generations (G1–G4) in each sub-line reared on the novel WIL diet and chicken feed control (CF). Lines include selectively bred lines (WIL1–WIL4), a within-line control (WILC), and a common control (CF). Each box represents the interquartile range, with the horizontal line indicating the median. Letters above boxes indicate statistically significant differences based on Tukey’s HSD post hoc comparisons following a two-way ANOVA of the line × generation interaction (*q* < 0.05). Black letters highlight significant differences between generations within each sub-line, while red and blue letters highlight significant differences between sub-lines within generations G2 and G3, respectively.

In the selection lines, larval weight generally peaked at intermediate generations (G2 or G3) before declining in G4 (Fig. 6). Two-way ANOVA revealed significant differences among sub-lines within generations, indicating a generation-dependent divergence in larval performance. At G2, CF and all size-selected lines (WIL1–WIL4) exhibited significantly higher larval weights than the dietary control WILC (*q* < 0.01), consistent with an early positive response to selection and/or diet adaptation. By G3, however, larval weight differed among selected sub-lines, with WIL2 and WIL4 showing significantly lower weights compared to CF and WIL1, and reduced performance relative to WILC. This increasing heterogeneity among sub-lines preceded the overall decline observed at G4.

To explore associations between larval weight and gut microbiota, Spearman’s rank correlation was performed using D10 samples across all generations within each line. Although several genera showed significant correlations with larval weight (Table S2), no consistent trends were observed across generations (Fig. 5B), except in WIL1. In this line, *Brevibacillus*, *Caldifermentibacillus*, *Heyndrickxia*, *Lysinibacillus*, and *Paenibacillus* were significantly more abundant in G4 than in G3 (*q* < 0.05), whereas *Scrofimicrobium* was higher in G2 than in G3 (*q* = 0.045). Several of these genera*—Brevibacillus*, *Caldifermentibacillus*, *Heyndrickxia*, and *Paenibacillus*—also showed negative correlations with larval weight, aligning with the observed performance decline in G4. However, the absence of comparable patterns in other lines limits broader inference of a microbiota-mediated effect on larval weight.

### Species-level shifts in BSFL gut microbiota across generations

To refine genus-level patterns, species-level differences were examined at D0 and D10 within each line across generations (Fig. 5C). Consistent with genus-level results, most differentially abundant taxa were detected at D0, particularly in the selected lines. These shifts were largely line-specific and not conserved across developmental stages, with WIL1 being the only line showing significant differentiation at D10. Specifically, *Caldifermentibacillus hissashi* and *Paenibacillus cookii* were enriched in G4 relative to G3 (*q* < 0.05), paralleling genus-level observations.

In WIL2, *Bacillus amyloliquefaciens*, *B. subtilis*, *B. velezensis*, *Paenibacillus ottowii*, and *Providencia polymyxa* were differentially abundant between G3 and G4 and between G2 and G4, with lower abundance in G4 (*q* < 0.05). WIL3 exhibited a single differentially abundant species, *Providencia stuartii* (G2: 3.0 ± 2.5% vs G3: 1.9 ± 1.4%; *q* = 0.042). WILC, which was not subjected to targeted selection, showed limited shifts confined to early developmental stages (D0) between G3 and G4. These included several *Enterococcus* species (*E. canintestini*, *E. faecalis*, *E. faecium*, *E. innesii*, *E. larvae*, *E. mediterraneensis*, *E. olivae*, and *E. saccharolyticus*; *q* < 0.05).

### Deviations from neutral assembly in the gut microbiota

To investigate whether shifts in gut microbial communities across selectively bred BSF lines were driven by neutral or deterministic processes, the Sloan neutral community model (SNCM) was applied to species-level OTU data. This model evaluates the relationship between each taxon’s mean relative abundance in the metacommunity and its frequency of detection in local communities. Taxa were classified as “Neutral,” “Above” (more frequently detected than predicted), or “Below” (less frequently detected than predicted) using 95% Wilson score confidence intervals.

Model fits were moderate across all six sub-lines (*R*² = 0.35–0.49; migration rates *m* = 0.0343–0.0586), indicating that stochastic processes, such as random dispersal, contributed to community assembly but accounted for only 35–49% of observed variation (Table S3). Most taxa conformed to neutral expectations, while selectively bred lines (WIL1–WIL4) displayed the highest number of “Above” taxa (240–267) and substantial numbers of “Below” taxa (110–122), suggesting enrichment of potentially beneficial microbes and exclusion of taxa incompatible with the evolving gut environment. By contrast, CF, maintained on standard feed without selection, had the lowest richness (*n* = 1,213) and few deviations from neutrality (Above: 204; Below: 105), reflecting a more stable, baseline microbiota. WILC, unselected but maintained on a novel diet, showed the highest richness (*n* = 1,663) and more “Below” taxa (*n* = 132), indicating a measurable effect of dietary stress on community assembly (Table S3).

**Fig 7:**
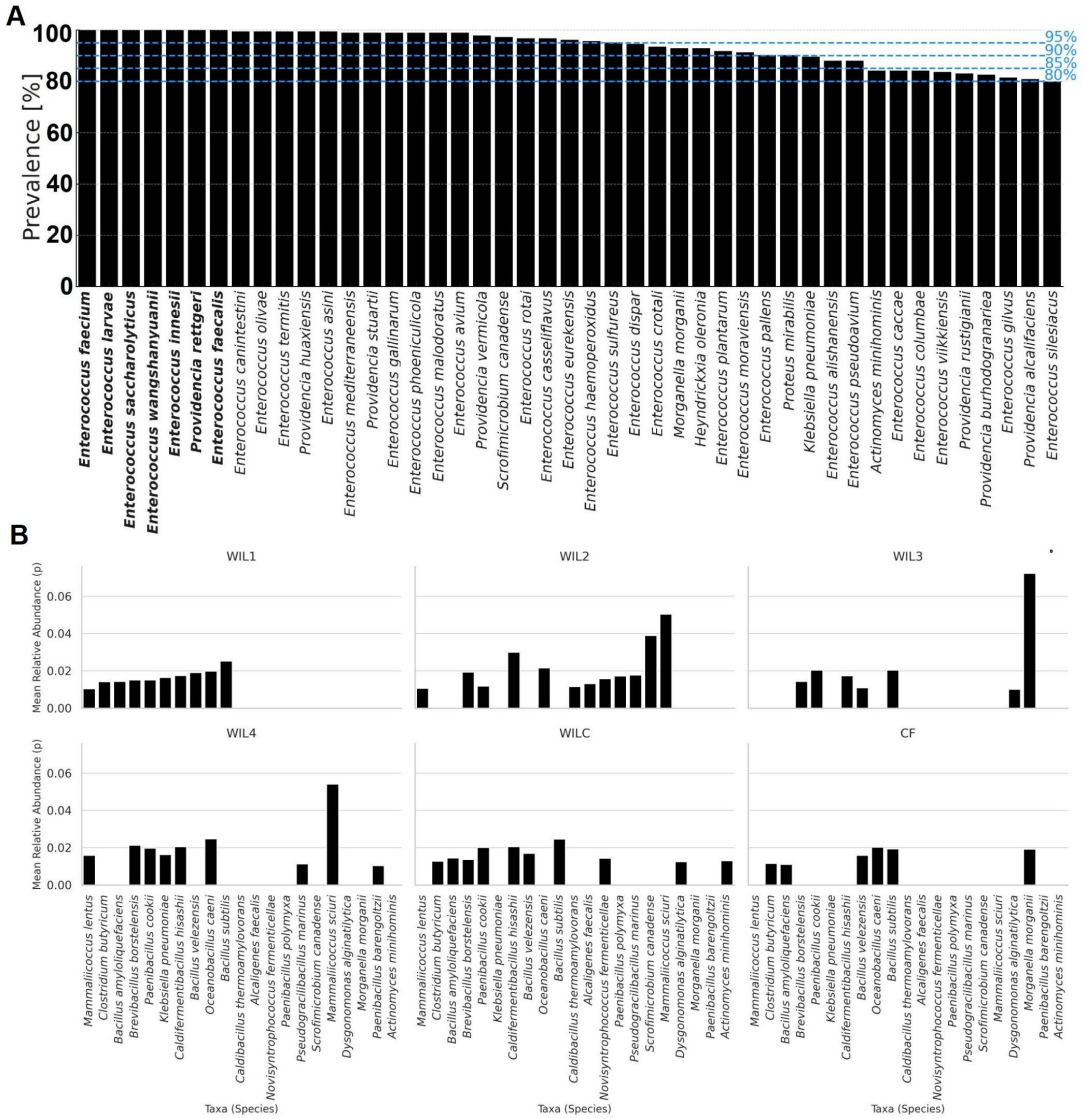
Prevalence of shared bacterial species across all BSF individuals, irrespective of diet, developmental stage, or selective pressures (**A**). Dashed horizontal lines indicate prevalence thresholds (80%, 90%, 95%, and 100%) used to define core taxa. (**B**) Sloan neutral model classification of taxa falling below neutral expectations after stringent filtering, shown for each sub-line. The y-axis represents the mean relative abundance of each taxon within the respective sub-line.

Several taxa were present in 100% of the samples, including *Enterococcus faecium*, *E. larvae*, *E. saccharolyticus*, *E. wangshanyuanii*, *E. innesii*, *Providencia rettgeri*, and *E. faecalis*, representing a highly conserved species-level core (Fig. 7A). Most of these core taxa were classified as “Neutral” or “Above,” suggesting either stochastic persistence or selective advantage (Table S4). Conversely, stringent thresholds identified 21 taxa consistently “Below” predictions (Fig. 7B), including *Brevibacillus borstelensis*, *Paenibacillus cookii*, and *Caldifermentibacillus hisashii*, highlighting species consistently excluded across WIL diet-fed lines. Other underrepresented taxa included *Morganella morganii*, *Actinomyces minimnominis*, *Dysgonomonas alginatilytica*, and *Bacillus velezensis* in multiple sub-lines.

## DISCUSSION

This study investigated the transgenerational effects of a novel diet on larval weight and gut microbiota in BSFL derived from a single genetic population. Despite shared ancestry, sub-lines exposed to the same dietary treatment exhibited divergent trajectories in larval performance and gut microbial composition across four generations. These findings underscore the dynamic interplay between environmental pressures and microbiota-mediated plasticity, demonstrating that significant phenotypic and microbial changes can emerge independently of host genetic differentiation.

Larval weight trajectories followed a consistent pattern across all sub-lines, increasing until G2 or G3 before declining sharply at G4. This decline occurred not only in the lines subjected to selection on larval size (WIL1–WIL4) but also in the non-selected controls (WILC and CF), indicating that the G4 decline was not driven solely by directional selection. These phenotypic trends corresponded with the low narrow-sense heritability estimate for larval weight (*h²* = 0.206), suggesting that genetic contributions to the trait were modest within this population. Instead, environmental or microbial factors, alongside potential physiological trade-offs, likely exerted greater influence on larval performance outcomes. Previous studies examining *Musca domestica* and BSFL have documented similar shifts in energy allocation from growth to maintenance functions in response to environmental stress, particularly under prolonged adaptation to novel substrates (16, 22).

### Transgenerational plasticity in larval gut microbiota

Bacterial shifts were evident at both temporal (between larval ages/metabolic phases within a generation) and transgenerational (across generations for a given larval age) scales. Most strikingly, age-related changes in community composition became increasingly pronounced in later generations (G3 and G4). For instance, *Scrofimicrobium*, a genus previously associated with nutrient extraction from complex organic substrates (27), was consistently enriched at D5 during later generations across most lines. An increase in *Paenibacillus* abundance, accompanied by a decrease in *Providencia*, was observed in several lines. Previous work has linked *Paenibacillus* to reduced fat conversion efficiency and *Providencia* to enhanced larval weight gain (15), suggesting that these shifts reflect a compensatory microbial response to dietary stress during peak larval metabolism, wherein the larvae prioritize nutrient acquisition over energy storage. Metabolically active genera, including *Enterococcus*, *Klebsiella*, and *Bacillus* (28), fluctuated significantly across the WIL-fed lines, suggesting that microbiota responses occurred in a sub-line-specific manner, despite exposure to the same novel substrate.

### Gut microbiota shift in early development

Transgenerational comparisons of each larval age revealed that the developmental stage D0 exhibited the most consistent changes across generations, suggesting that microbiota composition was primarily influenced early in larval development. Specifically, *Bacillus* and *Paenibacillus* were enriched at D0 in G2–G3 but declined in G4, while *Klebsiella* became highly abundant at G4. These trends likely reflect a shift in microbial functional roles rather than community destabilization. Additionally, *Brevibacillus*, *Heyndrickxia*, and *Caldifermentibacillus*, genera associated with fermentation and breakdown of complex carbohydrates (29–31), were enriched in G4 D10 samples, particularly in WIL1. Although these taxa negatively correlated with larval weight in the WIL lines, their enrichment may represent an adaptive response wherein the microbiota reallocates functional investment from growth promotion to digestive efficiency under prolonged dietary stress, prioritizing nutrient extraction over biomass accumulation. This pattern could reflect a potential trade-off strategy of resource allocation shifts toward resilience and survival rather than growth (19).

Particularly, differentially abundant taxa were largely absent in D10 samples across most other sub-lines, despite the observed G4 performance decline, supporting the hypothesis that microbial influences on larval phenotype may be determined during early developmental windows. In G4 larvae, immune-modulatory species such as *Providencia stuartii* and *Klebsiella pneumoniae* were enriched (32–34). A previous study isolating *K. pneumoniae* from the gut of *Bombyx mori* demonstrated high β-endoglucanase and α-amylase activity, indicating a capacity to degrade plant cell walls (35). Given that the novel WIL diet contains palm kernel meal, a substrate rich in lignocellulosic material, the increased abundance of *K. pneumoniae* in later generations may reflect an adaptive microbial shift toward enhanced fiber degradation under prolonged dietary exposure. In contrast, beneficial growth-associated taxa, such as *Paenibacillus polymyxa* and *Bacillus subtilis*, were notably reduced, suggesting a microbial reorganization away from growth promotion and towards digestion-oriented strategies in response to sustained dietary stress (36, 37).

These findings suggest that microbial restructuring may be closely tied to host-driven physiological adjustments. As larvae face the nutritional constraints of the WIL diet, shifts in digestive and metabolic processes, such as altered enzyme production, immune regulation, and energy partitioning, could create selective pressures within the gut microenvironment that favor specific bacterial taxa. Future studies incorporating molecular-level analyses would be instrumental in disentangling these complex host–microbe interactions. For instance, integrating transcriptomic or metabolomic profiling with microbiome data would enable the identification of host genes and metabolic pathways associated with microbial shifts, offering mechanistic insight into how host physiology and gut microbiota co-evolve under dietary stress.

### Stochastic vs deterministic forces in gut microbiota assembly

The application of the SNCM revealed that while stochastic processes contributed to the assembly of the BSF gut microbiota, deterministic forces, such as dietary selection and/or host filtering, accounted for a substantial portion of the observed variation, potentially playing a dominant role in shaping the gut microbiota composition, particularly under sustained exposure to the novel WIL diet. The consistent presence of a core group of *Enterococcus* and *Providencia* species across all lines, genera previously identified as part of the core microbiota in wild-type BSF (15), was reaffirmed in this study. These species, including *Enterococcus faecium*, *Providencia rettgeri*, *Enterococcus saccharolyticus*, *E. larvae*, *E. innesii*, and *E. wangshanyuanii*, were all classified as either “Neutral” or “Above” neutral model expectations and were detected in all individuals, regardless of dietary or selective pressure. Their ubiquity suggests strong host compatibility and ecological persistence within the BSF gut environment. Although these taxa have not been functionally characterized in BSF to date, recent studies have begun to associate them with putative roles. For instance, *Enterococcus faecalis* has been linked to enhanced fecundity, egg-hatching rate, and larval growth (38). Notably, *E. faecium*, *P. rettgeri*, and *E. saccharolyticus* were also found enriched in BSF lines subjected to environmental stress, where they were hypothesized to contribute to cytoskeleton-related pathways and survival under inhibitory conditions such as antibiotic exposure (39–41). This suggests that certain core taxa may not only persist for growth and development but instead confer resilience to physiological or environmental stress aiding in survival and fitness.

Conversely, 21 taxa, including *Brevibacillus borstelensis*, *Paenibacillus cookii*, and *Caldifermentibacillus hisashii*, were consistently classified as “Below” neutral expectations across the WIL-fed lines. While “Below” status can indicate active exclusion or niche incompatibility under altered gut conditions, it may also arise from patchy occurrence. For instance, taxa may be abundant only at specific larval ages or in certain generations but absent in others, thereby lowering their overall prevalence. Given that the WIL diet is enriched in hemicelluloses (mannan and xylan) with relatively low cellulose content (42), the consistently “Below” designation of *P. cookii* across multiple lines, despite its documented endoglucanase activity (43), is unsurprising. This pattern likely reflects limited selective advantage for cellulolysis under these specific substrate conditions and ecological competition that may favor taxa with mannan-/xylan-degrading capabilities.

Interestingly, the control line CF, maintained on a standard chicken feed without larval size selection, displayed the lowest species richness and the fewest deviations from neutral expectations. This pattern indicates that under optimal rearing conditions, microbial assembly may be predominantly shaped by neutral processes with minimal ecological filtering (44). The WILC line, exposed to the novel diet without larval selection, showed both the highest species richness and an increased number of “Below” taxa, reflecting strong dietary filtering even in the absence of phenotypic size selection (44). The selectively bred lines (WIL1–WIL4) further amplified these trends, consistently exhibiting the highest number of “Above” taxa and substantial numbers of “Below” taxa, suggesting ongoing enrichment for taxa providing a competitive or functional advantage, while concurrently excluding others (44).

### Plasticity vs long-term adaptation

Despite observable microbial and phenotypic shifts, the consistent weight decline at G4 across all lines, including CF and WILC, supports the notion that plastic responses possess inherent limits, and that sustained performance may require a stable gut bacterial community structure or co-adaptive dynamics between host and gut microbiota (45).

Crucially, the limited effective population size (~2000 larvae per generation) may have constrained the evolutionary potential of both the host and gut microbiota. Insect systems, such as *Drosophila*, have demonstrated that small effective populations were prone to microbial drift, stochastic loss of beneficial symbionts, and reduced resilience to environmental stressors (45). Similarly, a wild-caught BSF population established from ~ 2,000 individuals reported the greatest loss of genetic diversity by the second generation due to founder effects and continuous losses with successive generations from cumulative inbreeding and allele fixation in a small effective population size (9, 17). In addition, such losses in genetic diversity positively correlated with a decline in oviposition rate, egg hatchability rate, and prepupal weight by the fourth generation severely impacting larval fecundity (9).

Therefore, it is essential for industrial BSF breeding programs to account for both genetic and microbial factors influencing long-term transgenerational adaptation. Although BSFL exhibit remarkable dietary flexibility (16), successful adaptation to novel waste streams may depend on maintaining large, genetically and microbially diverse populations across multiple generations. Without sufficient genetic and microbial support, colonies may suffer from microbiota instability, reduced productivity, and inbreeding depression. These findings carry practical implications for long-term colony sustainability and underscore the importance of integrating microbiota monitoring into industrial breeding strategies for BSF.

### Conclusion

This study demonstrates that transgenerational exposure to novel diets can generate divergent phenotypic and microbial trajectories, even within genetically similar populations. While initial improvements in larval performance may be driven by phenotypic plasticity or microbial shifts, sustaining these gains over generations requires coordinated host-microbiota adaptation. In the absence of such coordination, particularly under small effective population sizes, performance declines, posing challenges for industrial BSFL production. These findings underscore the need for integrative breeding programs that account not only for host traits but also for microbial dynamics and long-term evolutionary potential. Future expansion of BSFL on novel waste substrates demands a sustainable approach that prioritizes the maintenance of both genetic and microbial diversity, ensuring resilience and performance stability in large-scale industrial systems.

## MATERIALS AND METHODS

### Heritability of BSF larval weight

Wild-caught BSF were used to establish a stock population WT, which had a low observed inbreeding coefficient (FIS = 0.121) prior to the commencement of the experiment (6). Narrow-sense (additive) heritability (*h*^2^) of pupal weight was estimated using a single-generation parent–offspring regression approach. Fifty 5-day-old WT larvae were randomly selected and transferred into a plastic container containing the WIL diet, composed of 50% palm kernel meal (PKM) and 50% okara (OKA) (sourced from Wilmar International Pte Ltd.) with a moisture content of 70%. Upon pupation, each larva was individually weighed and isolated in separate *Drosophila* breeding plastic tubes to prevent uncontrolled mating.

After adult emergence, individuals were sexed, and each was retained in isolation for an additional 4 days to allow females to reach reproductive maturity. The heaviest male and female were selected and introduced into mesh mating cages (each pair per cage, total 15 pairs) containing a corrugated cardboard strip suspended above an oviposition attractant composed of chicken feed and frass. Cardboard strips with deposited eggs were removed and suspended above fresh chicken feed in a new container, allowing hatched larvae to drop directly onto the feed.

Five-day-old progeny were collected and subjected to the same treatment as the parental generation. Both parental and progeny individuals were weighed at the pupal stage. Mid-parent pupal weight was calculated as the average of the male and female parental weights for each mating pair. The mean pupal weight of the progeny from each pair was then calculated from 10 pupae derived from per mating pair and used for heritability estimation.

Narrow-sense heritability was estimated using parent–offspring regression following the approach described by Falconer and Mackay (46), based on the equation:


$$h^{2}=b_{op}$$

where *b*_op_ represents the slope of the regression of offspring phenotype on mid-parent phenotype. For each family, the mean pupal weight of the offspring cohort was regressed against the average weight of both parents (mid-parent value). The slope of the best-fit linear regression line (*h*^2^) was taken as an estimate of narrow-sense heritability, representing the proportion of phenotypic variance attributable to additive genetic variance. The coefficient of determination (*R*^2^) was also calculated to indicate the proportion of variation in progeny weight explained by the mid-parent weight.

Linear regression was performed in R (version 4.1.2) using the lm() function, with mid-parent pupal weight as the independent variable and mean progeny pupal weight as the dependent variable. A total of eight families were included in the analysis.

### Experimental set-up

Following the heritability estimation, a large-scale diet experiment was conducted to investigate the transgenerational response of larval size and gut microbiota to a novel substrate. The WT founder population was divided into six sub-lines: CF, WILC, WIL1, WIL2, WIL3, and WIL4, each with three replicates. The CF sub-line functioned as a general control (no selection) and was reared on a standard chicken feed (PK Agro-industrial Products M Sdn Bhd; Johor, Malaysia), whereas WILC functioned as dietary control (no selection) and was reared on a novel diet (WIL diet) composed of 50% palm kernel meal (PKM) and 50% okara (OKA). The WIL1 to WIL4 sub-lines were subjected to selection for increased larval size on the WIL diet. Each replicate of these sub-lines was initiated with ~2,000 WT larvae and reared on their respective diets provided *ad libitum* (total wet weight of diet: 6.7 kg per tray). On day 10 of the experiment, when more than 50% of larvae had reached the prepupal stage, individuals were separated from the residual substrate using a two-step sieving process. The first step involved an industrial sieve with a fine mesh (~1 mm), followed by manual sieving through a coarser mesh (~3 mm) to remove remaining residues. After these sieving steps, triplicate trays within each sub-line were pooled.

For the size-selected lines (WIL1–WIL4), a third sieving step (mesh size: ~5 mm) was applied to separate larger larvae/prepupae from smaller individuals, from which the top 1,000 largest individuals were selected for mating. The CF and WILC sub-lines underwent no size selection; instead, 1,000 larvae/prepupae were randomly chosen for mating. This process was repeated over three successive generations (G2 to G4) to monitor phenotypic response and gut microbiota shifts under diet-mediated selection pressure.

### Sample collection and gut isolation

At the start of the experiment (D0), WT larvae were collected and snap-frozen on dry ice. For each sub-line (CF, WIL1- WIL4, and WILC), 10 larvae were collected from each triplicate on day 5 (D5) and day 10 (D10), weighed, and snap-frozen to assess temporal changes in gut microbiota composition. Sampling was conducted for each generation (G1–G4), and all collected samples were stored at −80°C until further processing.

The dissection procedure followed the technique outlined in previous studies (15, 47). Three whole larval guts per replicate were excised and pooled together to ensure sufficient material for subsequent DNA extraction. Before and after each excision, 70% ethanol was used to sterilize all surfaces and instruments. Isolated gut samples were stored at −80°C until further processing.

### Microbial community profiling

DNA was extracted from isolated gut samples using the QIAamp PowerFecal Pro DNA Kit (QIAGEN) following the manufacturer’s protocol with the vortex adapter for tissue homogenization. Before the final centrifugation step, 50 µL of C6 solution was added to the spin column membrane and incubated for 5 min at room temperature to ensure complete DNA elution.

Polymerase chain reaction (PCR) amplification was done targeting the full-length 16S rRNA gene (V1–V9 regions) using custom-designed barcoded 27F and 1492R primer pairs (Table S1) with unique barcode combinations for multiplexing. Ten microliters of Q5 High-Fidelity DNA Polymerase (New England Biolabs, Singapore), 1 µL of forward and reverse pre-mixed primer pairs (10 µM) with unique barcode combinations each, 50 to 100 ng DNA template, and water to reach a final volume of 25 µL were used. Each reaction was set up in triplicates to minimize amplification bias, and no template controls were included to ensure absence of contamination. The PCR cycling conditions were as follows: denaturation for 2 min at 95°C, 30 cycles of 20 s at 95°C, 20 s at 55°C, 1 min at 72°C, followed by final extension at 72°C for 7 min (Bio-Rad T100 Thermal Cycler). Verification of successful amplification was conducted through gel electrophoresis. PCR amplicons (~1,500 bp) were quantified and pooled in equimolar concentrations to generate seven libraries, each comprising 26 samples. These pooled libraries were subsequently prepared and barcoded using the Native Barcoding Kit 24 V14 (Oxford Nanopore Technologies), following the manufacturer’s instructions. Final libraries were loaded onto MinION Flow Cells (R10.4.1, FLO-MIN114) and sequenced using the Oxford Nanopore Technologies MinION platform.

### Nanopore bioinformatics

Following sequencing on the Oxford Nanopore platform, raw reads were basecalled and were first demultiplexed using Guppy to separate libraries based on native barcodes (NBD). Subsequently, demultiplexed reads from each library were subjected to a second round of demultiplexing and barcode trimming using a custom MATLAB script based on barcoded primers appended to individual samples. Sequencing reads in FASTQ format were imported and scanned for sample-specific barcodes provided in an accompanying barcode mapping file.

Demultiplexing was performed by detecting matching barcode sequences within the first and last 100 base pairs of each read. Reads were assigned to a specific sample only if both forward and reverse barcodes were identified. Once assigned, barcode regions were trimmed. Additional filtering was applied to retain reads within the size range of 1,000 to 2,000 base pairs to ensure high-quality, near-full-length 16S rRNA sequences.

Basecalled and demultiplexed reads exported as individual FASTQ files per sample were uploaded to the EPI2ME platform (version 2.11.0, Oxford Nanopore Technologies) for taxonomic classification using the Metagenomics Workflow. Within this pipeline, reads were aligned against the reference database using Minimap2 (version 2.26-r1175) as the classification engine. Taxonomic assignments were resolved down to the species level. The pipeline generated raw read count tables and diversity metrics, including Shannon diversity index values, which were exported for downstream statistical analysis.

### Statistical analysis

#### Statistical analysis of larval weight

Larval weight on experimental day 10 (D10) was analyzed to assess generational differences within and between sub-lines. Statistical analysis was performed using R (version 4.1.2). A two-way Analysis of Variance (ANOVA) was used, with generation and sub-line included as fixed factors, to test for differences in larval weight and their interaction. When significant main effects or interactions were detected (α = 0.05), Tukey’s Honest Significant Difference (HSD) test was applied for post hoc pairwise comparisons. This method accounts for multiple comparisons by controlling the family-wise error rate, and adjusted *P*-values (*q* values) were used to determine significant differences. Results were visualized using ggplot2 (version 3.5.1).

#### Statistical analysis of 16S rRNA gene sequence data

Raw count abundance tables, taxonomic assignments, and α diversity metrics (Shannon index) generated via the Epi2Me platform were imported into RStudio (version 4.1.2) for downstream statistical analysis. All analyses were performed using a combination of R packages, including phyloseq, vegan, ggplot2, dplyr, tidyverse, and patchwork.

Shannon α diversity values were analyzed using pairwise Wilcoxon rank-sum tests between groups (sub-lines, generations, and larval age), with *P*-values adjusted using the Benjamini–Hochberg method (reported as *q* values). A three-way ANOVA was additionally performed to assess the main and interaction effects of sub-lines, generations, and larval age on Shannon α diversity. Boxplots were generated to visualize diversity changes over time and across experimental groups.

To evaluate differences in overall community structure within each sub-line, β diversity was assessed using principal coordinates analysis (PCoA) based on the Bray–Curtis dissimilarity metric, clustering with 95% confidence ellipses grouped by age (D0, D5, D10) and generation (G1–G4) (phyloseq package version 1.38.0).

Permutational Multivariate Analysis of Variance (PERMANOVA) was used to test for significant differences in microbial community composition among groups. Analysis was performed using the vegan package (version 2.6-8), with 999 permutations, applying a correction for multiple testing. The analysis evaluated the effects of sub-lines, larval age, and generation on microbial community composition in three contexts: (i) globally across all generations and time points within each line, (ii) pairwise comparisons of days within each generation and generations within each day, and (iii) comparisons including WT samples (G1, D0) as a baseline reference. To verify the assumption of homogeneity of group variances underlying PERMANOVA, beta dispersion analysis was performed using the vegan package on the same Bray–Curtis distance matrices. Pairwise tests of dispersion were applied only to group comparisons that yielded statistically significant PERMANOVA results.

Differential abundance analysis was conducted using the ALDEx2 package (version 1.26.0) to identify taxa whose relative abundances differed significantly among groups. Abundance data (with taxa <1% across all samples collapsed) were centered log-ratio (CLR) transformed and underwent Monte Carlo Dirichlet sampling with 128 Monte Carlo instances (mc.samples = 128). Statistical testing was performed using Welch’s *t*-test, and significance was determined based on adjusted *P*-values. Pairwise comparisons were conducted (i) between sub-lines, (ii) across time points within each generation, and (iii) across generations within each time point at both genus and species level. Heatmaps were generated using ggplot2 (version 3.5.1).

Spearman’s rank correlation coefficients were calculated using Microsoft Excel to examine the relationship between larval weight and gut bacterial taxa at both genus and species levels across sub-lines. Statistical significance was assessed using Student’s *t*-test, with adjustments for tied ranks applied using the Bonferroni correction method.

To evaluate the role of stochastic processes in shaping gut microbial community assembly across the selectively bred BSF lines, the SNCM was applied using a custom implementation based on the original sncm.fit() (48). The analysis was conducted in R using the packages minpack.lm, Hmisc, and stats4, with species-level OTU count data as input. Prior to model fitting, OTUs with zero counts across all samples within each sub-line (WIL1 to WIL4, WILC, and CF) were excluded.

The model fits the relationship between the mean relative abundance of each taxon (across all samples within a sub-line) and its frequency of detection (i.e., the proportion of samples in which it was present), estimating a migration probability parameter (*m*) via non-linear least squares. Ninety-five percent Wilson score confidence intervals (CI) were calculated around the model-predicted detection frequencies, and taxa were classified as “Neutral” if their observed frequency fell within the CI, “Above” if detected more often than expected, and “Below” if detected less often than expected.

Model fits were evaluated using *R*², root mean square error (RMSE), Akaike Information Criterion (AIC), and Bayesian Information Criterion (BIC). Following taxonomic assignment, between 1,139 and 1,285 Neutral taxa, 237–268 Above-neutral taxa, and 113–138 Below-neutral taxa were identified across the six sub-lines. To identify taxa of high confidence and greater biological relevance, additional filters were applied in a stepwise manner. First, only taxa with relative abundance greater than 1% (*P* > 0.01) were retained to remove rare species. Next, only taxa classified as “Above” or “Below” were retained to focus on those deviating from neutral expectations. This was followed by a detection filter, retaining only taxa present in ≥20% of samples (freq ≥ 0.2) within each sub-line, to ensure consistency of detection. Finally, taxa were filtered based on absolute deviation from predicted detection frequency (freq.pred > 0.05) and retained, indicating a substantial deviation from neutral model predictions.

## Data Availability

Supplementary figures and tables are provided in the Supplemental material. The demultiplexed Nanopore 16S rRNA sequences used in this study are available at Zenodo: https://doi.org/10.5281/zenodo.18168208 (49). Demultiplexing scripts, analysis files, and R code are available on GitHub: https://github.com/ReproLab/Transgenerational-dynamics-of-BSF-gut-microbiota.

## SUPPLEMENTAL MATERIAL

Supplemental material spectrum.01903-25-s0001.docx Tables S1 to S4; Fig. S1 and S2.

ASM does not own the copyrights to Supplemental Material that may be linked to, or accessed through, an article. The authors have granted ASM a non-exclusive, world-wide license to publish the Supplemental Material files. Please contact the corresponding author directly for reuse.
