# Supplementary material for: Transgenerational dynamics of gut microbiota in black soldier fly larvae (Hermetia illucens) reared on a novel substrate
Source: Microbiol Spectr. 2026 Mar 31;14(5):e01903-25. doi: 10.1128/spectrum.01903-25 (PMC13141891; doi:10.1128/spectrum.01903-25)
Supplement: Supplemental material — Tables S1 to S4; Fig. S1 and S2. [file spectrum.01903-25-s0001.docx]

**Table S1.** Custom designed barcoded 27F and 1492R primers used in unique combinations for the study. Primers are written from 5’ to 3’ end. Text highlighted in green represents spacers, red represents barcodes and black represents the 16S rRNA gene sequence.

| **27F barcodes (5’ to 3’)** | | **Full 27F sequence including spacers (5’ to 3’)** |
| --- | --- | --- |
| F1 | AGGTTTGCTTAGAGTTTGAT | AAAAAGGTTTGCTTAGAGTTTGATYMTGGCTCAG |
| F2 | CTTTGGGCGTAGAGTTTGAT | AAAACTTTGGGCGTAGAGTTTGATYMTGGCTCAG |
| F3 | AGGCGCGTTTAGAGTTTGAT | AAAAAGGCGCGTTTAGAGTTTGATYMTGGCTCAG |
| F4 | GTTATTGTTGAGAGTTTGAT | AAAAGTTATTGTTGAGAGTTTGATYMTGGCTCAG |
| F5 | GCTGCTAACAAGAGTTTGAT | AAAAGCTGCTAACAAGAGTTTGATYMTGGCTCAG |
| F6 | ATGGATGTCTAGAGTTTGAT | AAAAATGGATGTCTAGAGTTTGATYMTGGCTCAG |
| F7 | CCACATTTAGAGAGTTTGAT | AAAACCACATTTAGAGAGTTTGATYMTGGCTCAG |
| F8 | GTCAGGGCGGAGAGTTTGAT | AAAAGTCAGGGCGGAGAGTTTGATYMTGGCTCAG |
|  | **1492R barcodes (5’ to) 3’** | **Full 1492R sequence including spacers (5’ to 3’)** |
| R1 | AGGTTTGCTTACGG | AAAAAGGTTTGCTTACGGYTACCTTGTTACGACTT |
| R2 | CTTTGGGCGTACGG | AAAACTTTGGGCGTACGGYTACCTTGTTACGACTT |
| R3 | AGGCGCGTTTACGG | AAAAAGGCGCGTTTACGGYTACCTTGTTACGACTT |
| R4 | GTTATTGTTGACGG | AAAAGTTATTGTTGACGGYTACCTTGTTACGACTT |
| R5 | GCTGCTAACAACGG | AAAAGCTGCTAACAACGGYTACCTTGTTACGACTT |
| R6 | ATGGATGTCTACGG | AAAAATGGATGTCTACGGYTACCTTGTTACGACTT |
| R7 | CCACATTTAGACGG | AAAACCACATTTAGACGGYTACCTTGTTACGACTT |
| R8 | GTCAGGGCGGACGG | AAAAGTCAGGGCGGACGGYTACCTTGTTACGACTT |

**Table S2.** Spearman’s Rank coefficient was calculated to explore the correlation between larval weight and gut bacterial taxa at the genus level on experimental day 10. A positive value indicates a positive correlation, while a negative value indicates a negative correlation. Significance of correlating parameters was determined using Student’s t-test followed by calculation of *p*-values (levels of significance are indicated by asterisk(s)). Only significant taxa (*p* < 0.05) are shown, with their corresponding Spearman’s Rank coefficients (R-values).

**Table S3.** Summary of Sloan neutral model fitting parameters for each BSF sub-line*.* The Table reports the estimated migration rate (m), 95% confidence intervals (CI) around the model predictions (lower and upper bounds), maximum likelihood estimate (MLE) of m, model fit statistics including R², root mean square error (RMSE), Akaike Information Criterion (AIC), and Bayesian Information Criterion (BIC), as well as species richness, and detection limit (minimum non-zero relative abundance) for each sub-line (WIL1–4, WILC, CF). Model fitting was performed using non-linear least squares with Wilson score confidence intervals around predicted detection frequencies. The columns “Above,” “Neutral,” and “Below” indicate the number of taxa whose observed detection frequencies fell above, within, or below the 95% confidence envelope of the model, respectively.

**Table S4.** Sloan neutral model classification of core gut bacterial species detected across all BSF sub-lines, diets, and generations. Species are categorised as Neutral (occurring at frequencies consistent with neutral assembly) or Above (occurring more frequently than expected under neutrality, suggesting selective enrichment).

|  | WIL1 | WIL2 | WIL3 | WIL4 | WILC | CF |
| --- | --- | --- | --- | --- | --- | --- |
| *Enterococcus larvae* | Neutral | Neutral | Neutral | Above | Neutral | Neutral |
| *Enterococcus faecium* | Above | Neutral | Neutral | Neutral | Neutral | Neutral |
| *Enterococcus saccharolyticus* | Neutral | Neutral | Neutral | Neutral | Neutral | Neutral |
| *Enterococcus faecalis* | Neutral | Neutral | Neutral | Neutral | Neutral | Neutral |
| *Enterococcus innesii* | Neutral | Neutral | Neutral | Neutral | Neutral | Neutral |
| *Enterococcus wangshanyuanii* | Neutral | Neutral | Neutral | Neutral | Neutral | Neutral |
| *Providencia rettgeri* | Neutral | Above | Neutral | Neutral | Neutral | Neutral |


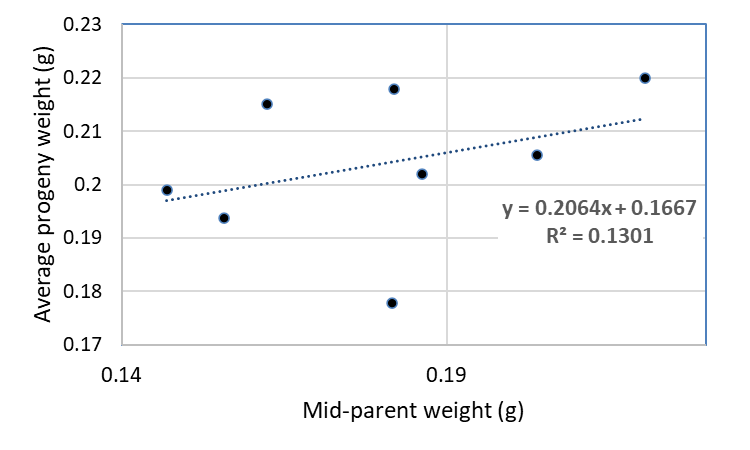
**Figure S1.** Parent-offspring regression plot based on average progeny weight (g) and mid-parent weight (g) across eight cages of *Hermetia illucens* WT population. The slope of the regression line estimates narrow-sense (additive) heritability (h^2^ = 0.2064), indicating a modest additive genetic contribution to weight variation. The coefficient of determination (R^2^ = 0.1301) shows that 13% of the variation in progeny weight is explained by mid-parent weight.


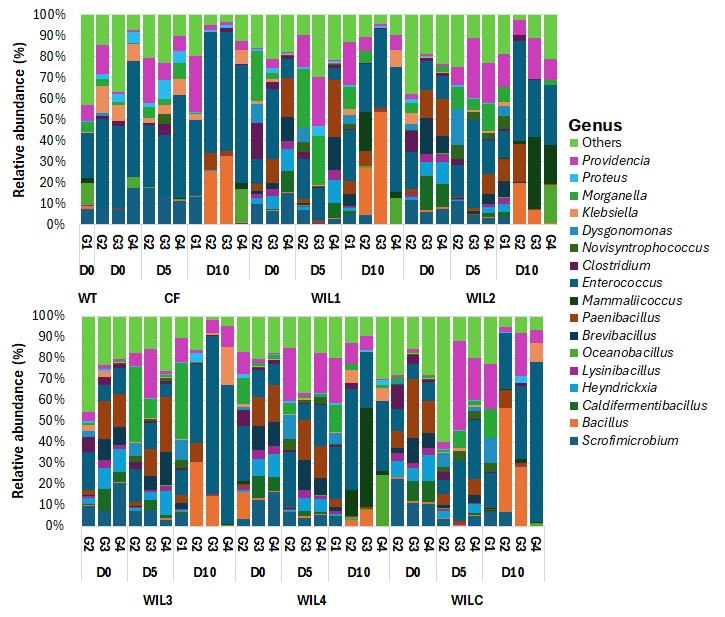


**Figure S2.** Relative abundances of genera found in the gut of BSFL under targeted selection for weight (WIL1 to WIL4) on a novel WIL diet as well as controls (CF and WILC). Top row displays the parent line (WT) from which generation one (G1), start of the experiment (D0) individuals were collected, as well as sub-lines CF, WIL1 and WIL2. The bottom row displays the rest of the sub-lines- WIL3, WIL4 and WILC. Individuals were categorised according to the age (D0, D5 and D10) followed by generations (G1 to G4). “Others” represents all remaining taxa, including those with < 1% relative abundance in the samples.
